# A nested parallel experiment demonstrates differences in intensity-dependence between RNA-seq and microarrays

**DOI:** 10.1093/nar/gkv636

**Published:** 2015-06-30

**Authors:** David G. Robinson, Jean Y. Wang, John D. Storey

**Affiliations:** 1 Lewis-Sigler Institute for Integrative Genomics, Princeton University, Princeton, NJ 08544, USA; 2 Center for Statistics and Machine Learning, Princeton University, Princeton, NJ 08544, USA; 3 Department of Molecular Biology, Princeton University, Princeton, NJ 08544, USA

## Abstract

Understanding the differences between microarray and RNA-Seq technologies for measuring gene expression is necessary for informed design of experiments and choice of data analysis methods. Previous comparisons have come to sometimes contradictory conclusions, which we suggest result from a lack of attention to the intensity-dependent nature of variation generated by the technologies. To examine this trend, we carried out a parallel nested experiment performed simultaneously on the two technologies that systematically split variation into four stages (treatment, biological variation, library preparation and chip/lane noise), allowing a separation and comparison of the sources of variation in a well-controlled cellular system, *Saccharomyces cerevisiae*. With this novel dataset, we demonstrate that power and accuracy are more dependent on per-gene read depth in RNA-Seq than they are on fluorescence intensity in microarrays. However, we carried out quantitative PCR validations which indicate that microarrays may demonstrate greater systematic bias in low-intensity genes than in RNA-seq.

## INTRODUCTION

Since the introduction of RNA sequencing (RNA-Seq) for measuring mRNA expression, one important question has been how the technology compares to microarrays in power and accuracy. Experiments have been carried out to compare microarrays and RNA-Seq, with some concluding that RNA-Seq shows greater power, accuracy and dynamic range (1,2) and others challenging that conclusion (3,4).

**Figure 1.**
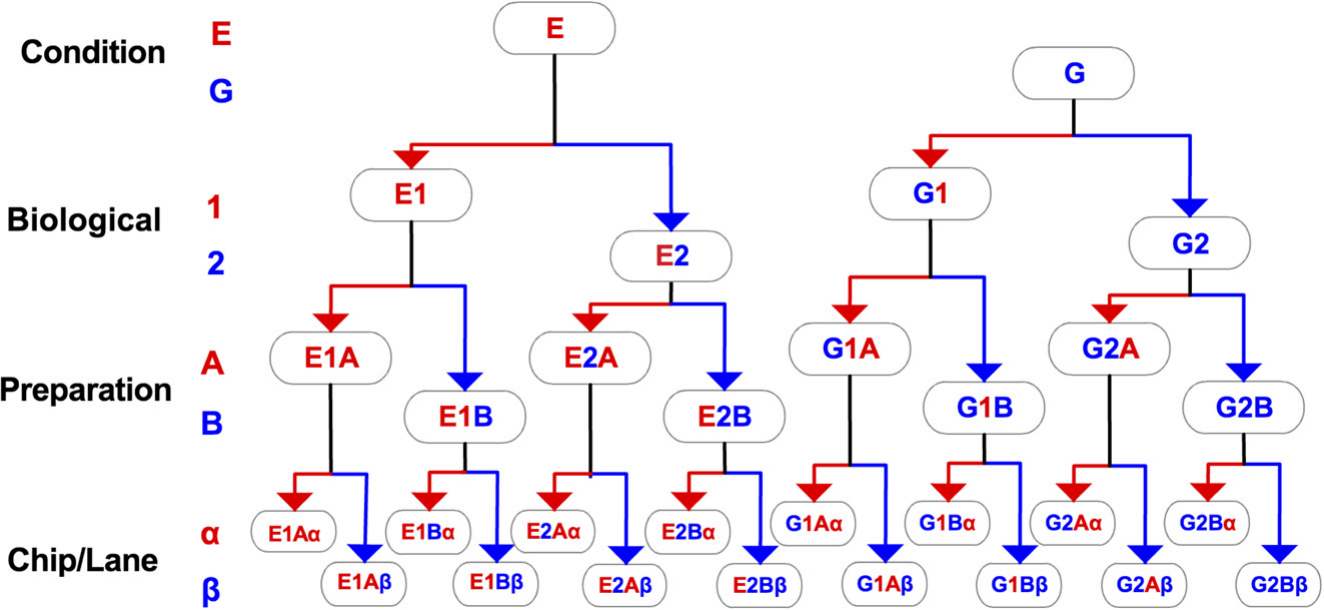
Schematic of the experiment. The Condition and Biological Replicate steps were performed irrespective of technology and these materials were then utilized in a technology-specific manner (microarrays or RNA-seq) for the Preparation and Chip/Lane steps.

We carried out a genome-wide gene expression experiment in a controlled setting on the yeast *Saccharomyces cerevisiae* in such a manner that the major sources of profiling variation can be unbiasedly partitioned and quantified (Figure 1). A single extraction of mRNA from each sample was quantified by both microarrays and RNA-seq in parallel. We multiplexed each lane of RNA-seq profiling so that it exactly mirrored the eight-array per chip design of the microarray platform that we utilized. This experiment allowed a direct and completely parallel investigation into the quantitative operating characteristics of RNA-seq gene expression profiling versus microarrays.

We use this experiment to examine the variation added by the RNA-Seq and microarray technologies. Our analysis utilizes two informative strategies: decomposing the variation contributed by each technology into multiple stages, which is made possible by the nested design and analyzing the variation as a function of gene expression intensity, which is known to influence technology-specific variation in both microarrays (5,6) and RNA-Seq (7–9). (Throughout this paper, we utilize ‘intensity’ of a gene as a term for both fluorescence intensity in microarrays and per-gene read depth in RNA-seq, both of which are measurements of a gene's abundance subject to technology-specific biases and sources of variation.) We find that the variance contributed by RNA-Seq is more intensity-dependent than that from microarrays, a result that is statistically significant and is robust across multiple normalization methods and *R*^2^ metrics. However, comparisons to quantitative PCR (qPCR) validations show that microarray may show systematic biases in low intensities, possibly due to cross-hybridization. This has implications for the design of future experiments.

A novel characteristic of our experiment relative to previous microarray to RNA-Seq comparisons is that we utilized barcode multiplexing to combine RNA-Seq replicates on each of two sequencing lanes, meaning that technical variation added by library preparation and handling is now distinguishable from ‘sampling’ variation added by the lane. Also notable is that our analysis takes into account the effect that intensity has on variation (9), which has confounded previous comparisons (10). We thus examine which technology adds more variation as a function of per-gene depth or intensity.

## MATERIALS AND METHODS

### Yeast growth and gene expression profiling

#### Growth of yeast in chemostats

The experiment was performed using yeast haploid strain DBY 12000 FY, a S288C derivative containing the wild-type HAP1 allele. A single colony was split into two overnight cultures, one containing ethanol (E) limitation medium with 60 C-mM (carbon millimolar) and the other containing glucose (G) limitation medium with 27 C-mM as the carbon sources (row 1 of Figure 1). One milliliter of each culture was used to inoculate a chemostat. After the chemostats reached stasis, 10 ml culture was collected and frozen at −80°C (see Supplementary Methods). A second set of biological samples were prepared the same way on a different day.

#### Microarray and RNA-Seq

RNA was harvested from each of the four samples (row 2 of Figure 1). The four RNA samples were then processed twice on different days using the Agilent Quick Amp Labeling kit (Part no. 5190-0424) to produce eight cRNA libraries, each of which was then hybridized on two separate chips (Yeast Expression 8 × 15K arrays) on different days according to the factorial design. After the arrays were washed and scanned, features were extracted using the Agilent Feature Extraction software to determine red and green intensities.

The same four RNA samples were also processed using the Illumina TruSeq RNA Sample Prep v2 LS protocol. Each sample was prepared twice up to the 3′ end adenylation step, then each of the eight preparations was split into two aliquots, after which each was indexed and amplified to complete the RNA-Seq library preparation (row 3 of Figure 1). The two groups of eight samples that were indexed together were each mixed at equimolar concentrations and sequenced on separate lanes on the same Illumina HiSeq 2500 flowcell, to produce 141 bp reads. In total, we produced profiles from 16 RNA-Seq samples and 16 microarray samples with identically nested experimental designs (row 4 of Figure 1).

#### Quantitative PCR

To perform qPCR, the four RNA samples were treated with RNase-Free DNase Set (Cat # 79254, Qiagen) column digestion to remove DNA in the samples. Total RNA was quantified using Quant-iT RNA assay Kit (Q33140, Invirogen) and Biotek Synergy Mx plate reader. A total of 750 ng total RNA was used in the first strand cDNA synthesis (SuperScript III Reverse Transcriptase, Cat # 18080, Invitrogen). Prevalidated FAM-MGB Taqman probes and primers mix for all candidate genes were ordered from Life Technologies. A total of 96-well plates (Cat# N8010560, ABI) and optical adhesive cover starter kit (Cat # 4313663, ABI) were used. qPCR reactions were set up by combining Taqman Gene Expression Master Mix (Cat # 4369016, Life Technologies) and individual probe plus primer mix. The 20-μl qPCR reactions were run on ABI 7900 HT Sequence Detection System using the following thermal protocol: 50°C, 2 min; 95°C, 10 min; 40 cycles of 95°C, 15 s and 60°C, 1 min; 95°C, 15 s; 60°C, 15 s; 95°C, 15 s.

Any candidate genes with more than one band on agarose gel after qPCR were excluded from further analysis. Three replicates of RT-qPCR for each RNA sample starting from cDNA synthesis to qPCR reaction were performed. Each measurement was normalized based on the average across all genes in a biological replicate and the log-fold change was estimated based on the difference in average number of cycles between ethanol and glucose samples.

### Preprocessing and statistical analysis

#### Normalization and differential expression

We used bowtie2 with the default set of parameters to map the RNA-Seq reads to the yeast genome and used htseq-count to match reads against the *S. cerevisiae* R64 release of the reference genome from the Sacchromyces Genome Database. Microarrays were normalized after averaging all preparation and chip replicates within each biological replicate to create two E replicates and two G replicates. The matrix of RNA-Seq counts was first pooled within preparation and chip replicates, then was transformed using voom from limma, which also computed precision weights for each observation (11). This pooling was necessary for differential expression because while the replicates at later nested stages introduced variation, they were not full biological replicates and treating them as replicates in the differential expression analysis would be pseudoreplication that underestimates the within-group variation (12). We tested for differential expression in each technology using a linear model with empirical Bayes shrinkage of t-statistics, implemented by limma version 3.24.0 (13).

#### Estimating contributions to variation

For calculations of the percent of variation explained, the microarray red/green log fold changes and the RNA-Seq log-transformed counts were compared, with the counts first transformed using voom (11). We compared multiple methods of normalizing both the microarray (MA) and RNA-Seq data (RS) between samples, using implementations in the normalizeBetweenArrays function in limma as well as the trimmed mean of M-values (TMM) (14) and relative log expression (RLE) (15) methods for RNA-Seq, but none made a qualitative difference in the resulting conclusions (Supplementary Figure S5).

For each gene, in both the normalized RNA-Seq or microarray data, we calculated the adjusted-*R*^2^, using a nested ANOVA on three linear models, which differed in which levels of the experiment were parameterized and which were left as residual variation (Supplementary Methods). The *R*^2^ estimates were then smoothed using LOESS across all genes based on their microarray intensity or RNA-Seq read depth.

## RESULTS

We carried out an experiment on both Agilent microarrays and Illumina RNA-Seq to investigate the effects of each source of variation on the inference of differential expression. We used the widely studied model organism *S. cerevisiae* to investigate differential expression associated with growth in different carbon sources, glucose (G) and ethanol (E), and we introduced steps to capture three additional factors or sources of variation. The sources of variation are: (i) Condition: biological condition of interest (G versus E); (ii) Biological Variation: natural biological variation between clonal populations; (iii) Preparation: sample handling and preparation; (iv) Chip/Lane: technical variation associated with either technology, such as array effects or lane effects.

Factors (i)–(iv) were sequentially nested, and at the stage of each nested factor, the sample was split such that each sample from the previous factor is balanced across both levels of the factor (Figure 1). Factors (i) and (ii) were performed only once to produce four samples of isolated RNA, while factors (iii) and (iv) were technology-dependent and therefore performed in parallel with microarrays and with RNA-Seq. We took advantage of a similar design on Agilent yeast microarrays (eight hybridizations per chip) and Illumina RNA-Seq (eight indexed samples per lane) to mimic the same approach across the two technologies, resulting in 16 microarray and 16 RNA-Seq profiles that show the amount of variation added at each stage. The RNA-Seq experiment achieved a depth of 170.8 million reads, with depths of 89.6 million and 81.1 million on each of the two lanes. The number of reads in each of the 16 samples is shown in Supplementary Table S1.

### Differential expression

The biological goal of this experiment is to infer differential gene expression in *S. cerevisiae* strain DBY12000 (S288c Hap1+ Mat *a*) cultivated in balanced growth conditions in chemostats using either glucose or ethanol as the sole carbon source (condition of interest). The chemostat device helped minimize any variations in growth conditions (such as physiological state, temperature, nutrient composition, etc.) so the study could directly interrogate the factor of interest: transcriptional responses to different carbon sources (16). We fit a linear model and calculated a moderated t-statistic to detect differential expression in each case, after log-transforming the RNA-Seq counts and fitting precision weights to each observation (11,13). We specifically tracked a biologically relevant set of 30 genes known to be involved in processes relevant to glucose or ethanol metabolism, namely gluconeogenesis, glycolysis, the tricarboxylic acid (TCA) cycle and the pyruvate branchpoint (17). The results of our differential expression testing for each gene in both microarray and RNA-Seq are shown in Supplementary Table S2.

**Figure 2.**
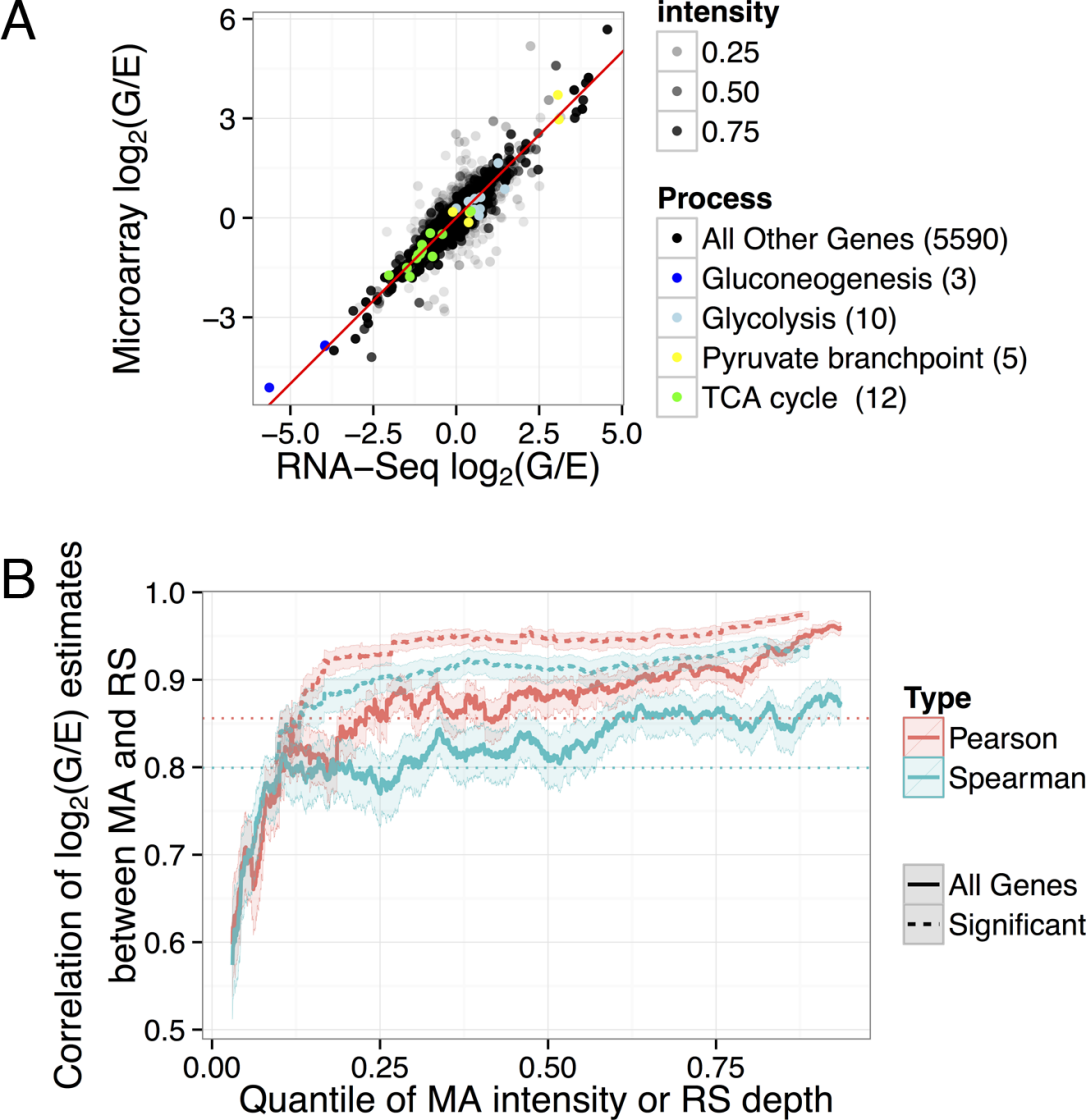
(**A**) Comparison between the log_2_(G/E) (log fold-change) estimates calculated from the RNA-Seq data (RS) or the microarray data (MA). The transparency of the points corresponds to the quantile of the intensity in microarray or RNA-Seq, whichever is lower. Thirty genes from biologically relevant pathways are highlighted in color. (**B**) Pearson or Spearman correlation of log_2_(G/E) estimate between microarray and RNA-Seq, within a 500 gene window rolling over microarray/RNA-Seq intensity ranking, shown with a 95% confidence interval (denoted by shaded regions). The dashed lines show the correlations for genes that were found to be significantly differentially expressed at FDR ≤1%. The correlation of all genes (0.856 Pearson, 0.799 Spearman) is shown as a horizontal dotted line.

One goal is to assess to what extent RNA-Seq and microarray experiments agree in their assessment of differential expression. The number of significant genes in each technology for various false discovery rate (FDR) thresholds, along with the number of genes that overlap, are shown in Supplementary Figure S1. We found that the fold-change estimates for differential expression showed a greater agreement (Spearman correlation of 0.799) than did the *P*-values (Spearman correlation of 0.546) and therefore focused on the fold change estimates for quantitative comparisons. Figure 2a compares the estimated log_2_(G/E) fold change ratios between the two technologies, with the opacity of each point determined by the quantile of the microarray intensity or RNA-Seq read depth, whichever is lower. We also identify the 30 biologically relevant genes highlighted in color. The microarray intensity of each gene was calculated as the average cy5 fluorescence intensity across all samples (where cy5 is the channel corresponding to the samples of interest), while RNA-Seq depth was calculated as the total number of reads mapping to the gene across all samples. (The relationship between the intensity quantile and the absolute intensity in each technology is shown in Supplementary Figure S2.) The correlation between the two technologies is highly dependent on read depth and microarray intensity, with the lowest-intensity genes exhibiting the greatest noise and therefore the lowest correlation.

Figure 2b shows how the Pearson and Spearman correlations between microarray and RNA-Seq effect size estimates depend on per-gene intensity, using a rolling window of 500 genes, ordered by intensity (again determined by the quantile of the microarray intensity or RNA-Seq read depth, whichever is lower). The Pearson correlation varies from 0.597 to 0.962, while the Spearman ranges from 0.574 to 0.884. The 30 genes in our biologically relevant set showed a 0.984 correlation of effect size estimates, which is understandable since almost all lie in the top 10% of read depth and microarray intensity. When only genes detected as differentially expressed in both technologies are considered, the correlation between the effect size estimates is higher, ranging from 0.810 to 0.975, but the intensity still has an effect on the agreement. The microarray and RNA-Seq assays identified as significantly differentially expressed 28 of the 30 biologically relevant genes at estimated FDR ≤5% (18), and the two technologies agreed on the direction of the change for all of these genes. This suggests that there is little difference between the two technologies in terms of estimating differential expression of high-intensity genes.

### Percentage of variation explained

**Figure 3.**
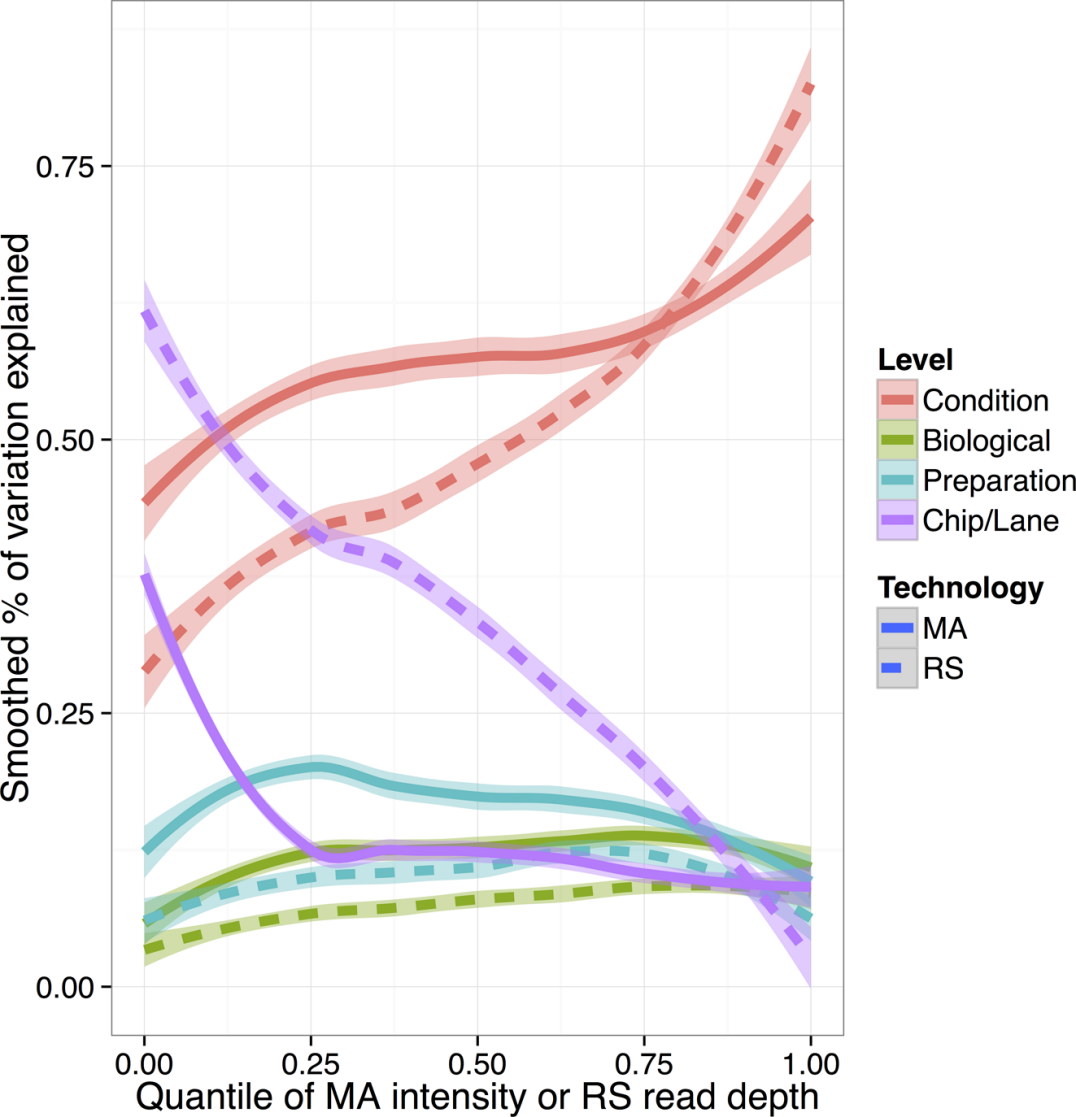
Percent of variance explained by each nested level of the experiment as computed by an ANOVA adjusted *R*^2^, and smoothed using LOESS across the intensity quantiles. Results from microarray data (MA) are shown in the solid lines and RNA-Seq data (RS) in the dashed lines. A 95% confidence interval is shown as the shaded region around each line.

The primary goal of the factorial experiment is to determine the relative amount of variation added at each stage of the experiment for each of the two technologies. Based on the correlation matrix (Supplementary Figure S3), the RNA-Seq and microarray assays easily distinguished between the ethanol and glucose samples and showed clustering within the chip/lane replicates, as expected from the experimental design. Any analysis should, however, consider the intensity-dependence of the variation that each technology contributes. We calculated the proportion of variation explained by the Condition, Biological and Preparation factors, as well as the residual variation due to the chip or lane, using a nested ANOVA analysis (‘Materials and Methods’ section). We computed this breakdown separately for each gene and smoothed the result across microarray intensity RNA-Seq depth using the local regression method, LOESS (Figure 3).

In both technologies at almost all intensities, the largest sources of variation were the treatment (ethanol versus glucose) and the chip/lane, in a tradeoff that depended strongly on intensity or read depth. The analysis indicated that the variance due to RNA-Seq lane at low-intensity genes was greater than that due to microarray chip. This difference is statistically significant: when the genes are divided into 10 bins based on depth or intensity, all bins up to the 80th percentile of intensity (less than a fluorescence intensity of 5500 or fewer than 30 000 reads) show a highly significant difference between the amount of variation added by the microarray chip versus the sequencing lane (Supplementary Figure S4). This difference would be costly to address by increasing the RNA-Seq read depth. One would have to triple the sequencing depth (from 171 million reads to 513 million reads total) for the percent of variance explained by condition for genes at the 25th percentile of RNA-Seq intensity (6500 reads per gene) to be comparable to genes at that percentile of microarray intensity. These conclusions were robust across multiple methods of microarray and RNA-Seq normalization (Supplementary Figure S5). To ensure that this discrepancy did not arise from the difference between the discrete count data from RNA-Seq and the quasi-continuous fluorescence intensity data from the microarray, we also calculated an alternative *R*^2^ designed for count data to determine the proportions of variance explained (Supplementary Methods) (19) and observed almost no difference (Supplementary Figure S6).

**Figure 4.**
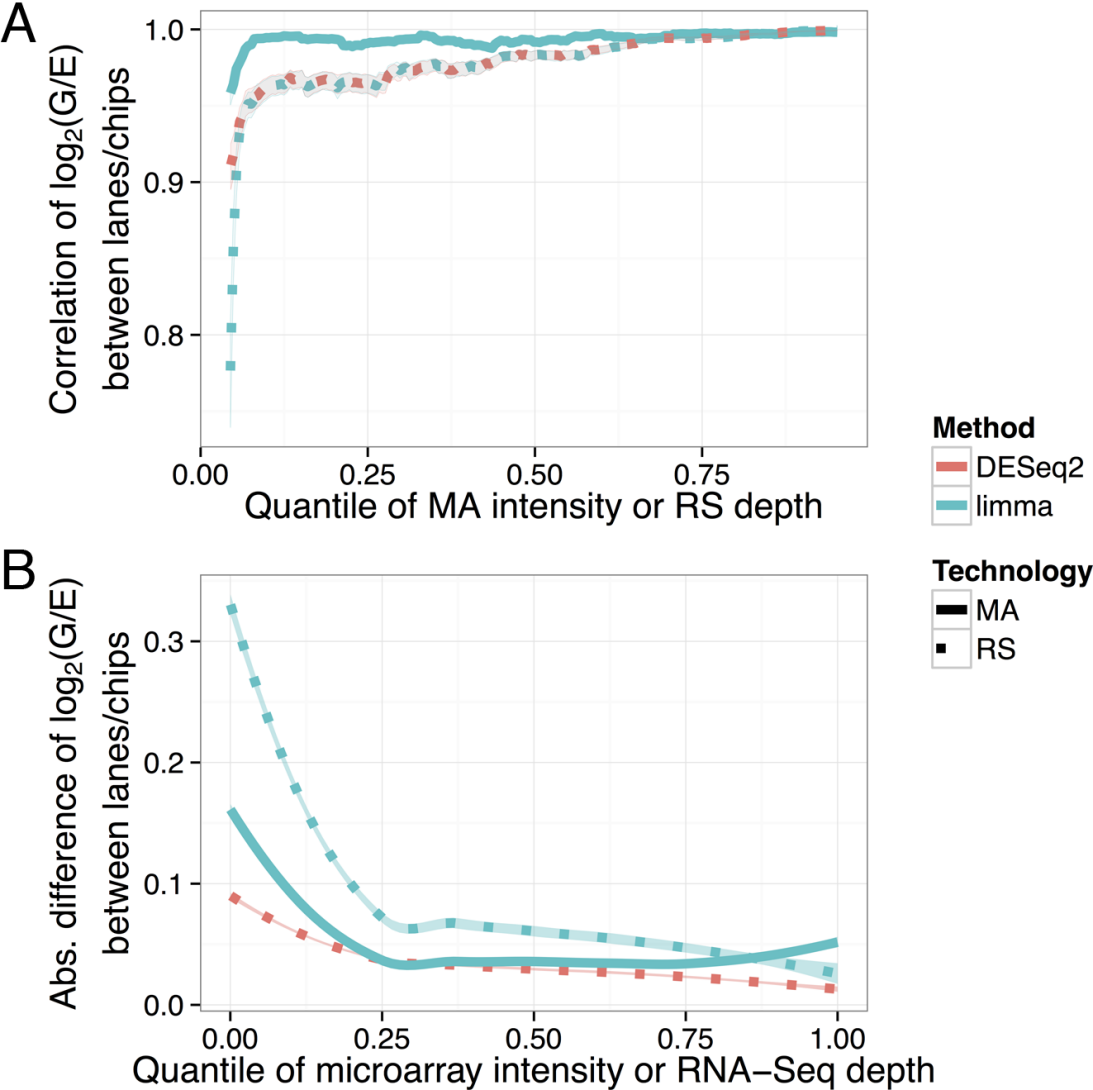
Comparisons of the log_2_(G/E) estimates within each technology, comparing the estimates computed for each of the two chips from the microarray data (MA) or two lanes from the RNA-Seq data (RS), using the same library preparations. (**A**) Pearson correlation between the two lanes of RNA-Seq or two chips of microarrays, within a 500 gene window rolling over intensity. (**B**) Absolute value of the log_2_(G/E) estimate difference between chips in microarray or lanes in RNA-Seq, smoothed using LOESS. Microarray is shown in the solid lines and RNA-Seq in the dashed lines. A 95% confidence interval is shown as the shaded region around each line (where the region may be thinner than the solid line itself).

To examine the variation added by the chip/lane level more directly, we also measured the difference in log fold change estimate when the same sample was run on two chips or two lanes. We computed the Pearson and Spearman correlation of log fold changes between chips or between lanes in overlapping windows of 500 genes ordered by intensity as above (Figure 4a), as well as a LOESS-smoothed curve of the absolute value of the difference (Figure 4b). Both analyses confirm that the difference between RNA-Seq lanes is more intensity-dependent than the difference between microarray chips, with a particularly great disagreement in low-intensity genes. One notable question is whether this effect can be mitigated by effect size shrinkage, such as the DESeq2 software, which is designed to improve the stability of estimates for low-depth genes (20). Figure 4 shows that DESeq2 causes the absolute difference in log fold changes to decrease, but does not substantially improve the correlation in any but the lowest-intensity windows. This suggests that effect size shrinkage can make estimates less variable between lanes, but does not remove the intensity dependence.

### Validation of low-intensity genes

**Figure 5.**
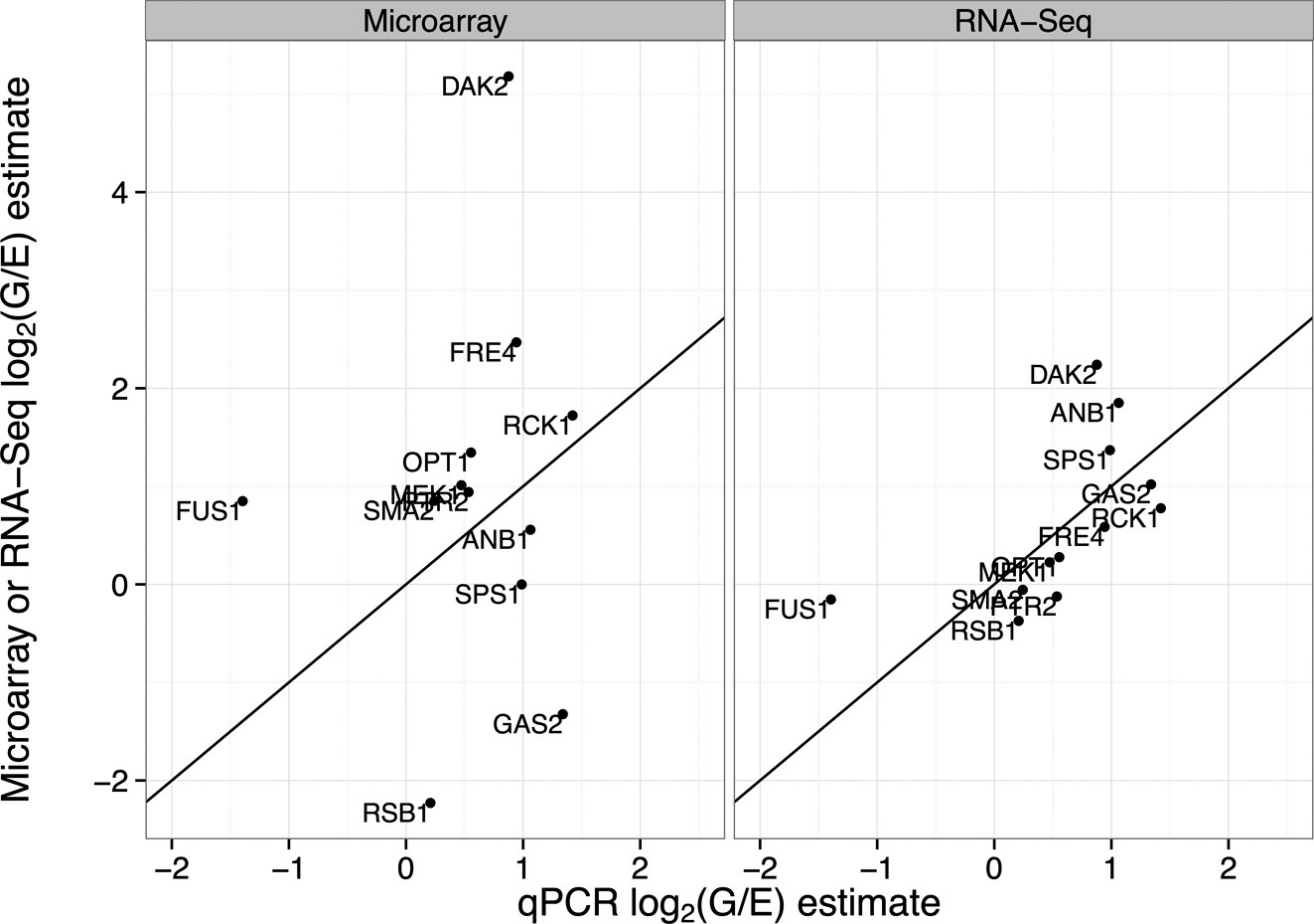
Comparison of the log fold change estimates measured with qPCR compared to estimates from the microarray or RNA-Seq, for 13 selected low-intensity genes that disagreed between RNA-Seq and microarray.

These results do not necessarily indicate that microarrays are more accurate at low intensities than RNA-Seq, only that they show greater consistency between replicates. Each technology may still possess biases that cause their measurements not to reflect the underlying mRNA abundance levels. To examine the accuracy of each technology more directly, we chose 13 genes in the bottom 20% of intensity for both technologies, for which the estimates of the log_2_(G/E) fold change disagreed by at least 1.0 between the technologies. On these low-intensity contested genes, we performed qPCR on the mRNA from the original experiment to provide an independent validation (‘Materials and Methods’ section). As shown in Figure 5, the qPCR results agreed much more closely with the RNA-Seq estimates than with the microarray: the Pearson correlation of estimated RNA-Seq and qPCR log fold changes is 0.671 (*P*-value 0.012), while the microarray to qPCR correlation is 0.024 (*P*-value 0.939).

**Figure 6.**
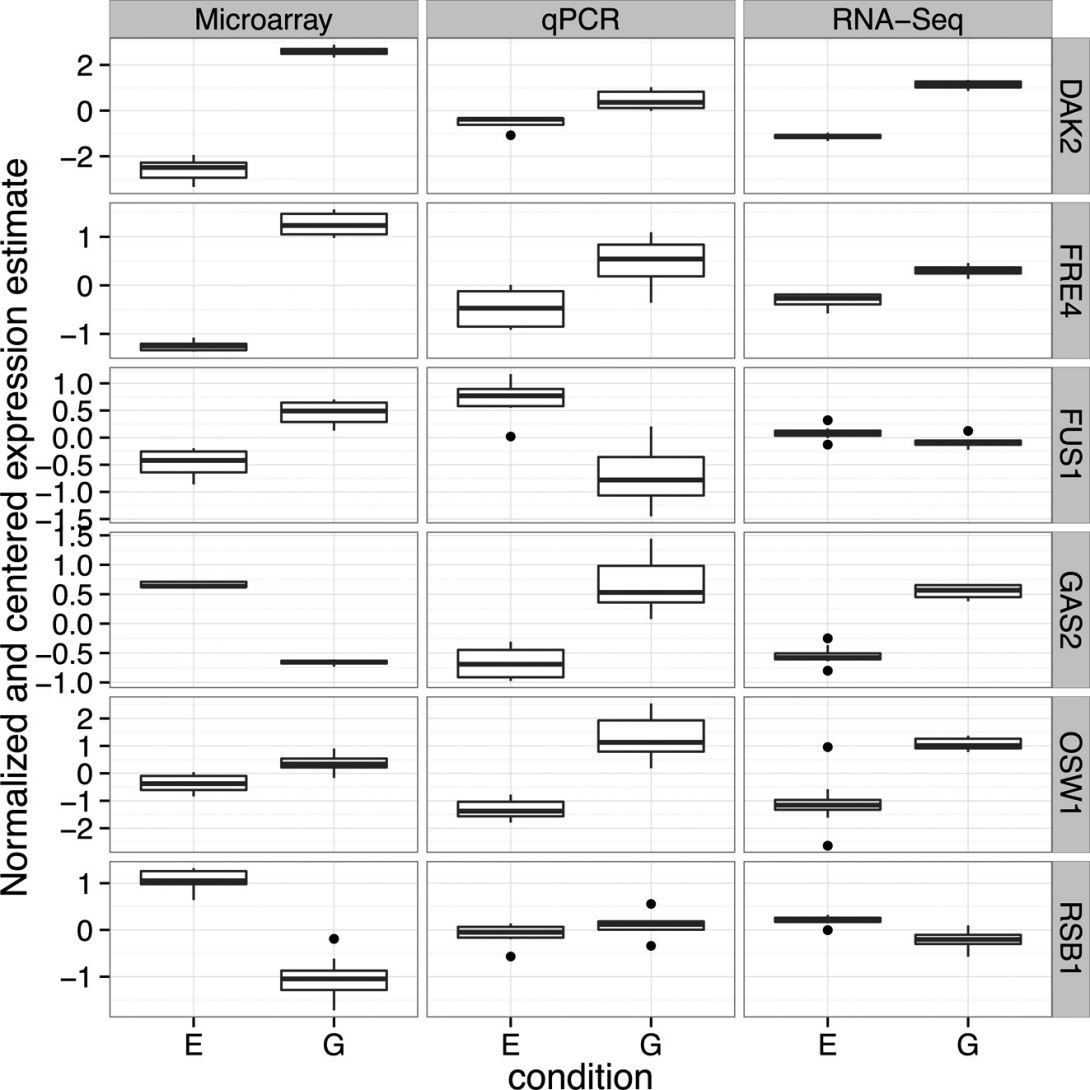
Boxplots comparing the normalized and centered expression values of microarrays, RNA-Seq and qPCR of the six genes for which microarray and qPCR most disagreed. This shows that in many cases, microarray measurements were very consistent between biological, technical and chip replicates. This suggests that the problem is not variation at low-intensity microarray measurements, but rather bias.

This leads to the question of whether the disagreement between microarrays and qPCR results from greater variation present at low-intensity, or rather systematic bias introduced by the technology, such as cross-hybridization or fold-change compression. Toward that end, we examined the distribution of normalized measurements across all biological, technical and chip/lane replicates from the six genes for which microarrays most strongly disagreed with qPCR (Figure 6). Even though these genes are low-intensity, in most cases the variation within microarray and RNA-Seq measurements was very small compared to the disagreement between the technologies. In an extreme example, GAS2 shows a reversed fold change relative to qPCR and RNA-Seq, but shows very little within-condition variation in any technology. Of these six genes, only OSW1 could plausibly be caused by fold change compression, since in other cases the microarray effect was greater than or a reversal from the RNA-Seq and qPCR observations (21). This suggests that the issue within these genes was not random variation present in low-intensity genes or fold change compression, but rather a bias that led to a spurious but statistically significant effect size estimate.

### Gene set enrichment

Many gene expression analyses seek to determine sets of genes whose expression changes within a particular condition in order to draw biologically relevant conclusions (22,23). We thus looked for enriched sets of genes within each technology using a Wilcoxon rank-sum test comparing genes within a gene set to those outside it (24,25).

Many enrichment tests look for genes that are unusually high or low on a ranked list, often ranked by statistical significance. However, ranking by statistical significance has been noted to lead to a confounding effect in RNA-Seq, where highly-expressed or high-depth gene sets are spuriously marked as significant (26,27). An alternative is to perform enrichment analysis on log fold change estimates, which would be expected to be less confounded with depth (28). To demonstrate this effect in our data, we performed gene set enrichment using either *P*-values or fold change estimates from each technology, then assigned the gene sets into five bins based on each gene set's median intensity (Supplementary Figure S7). We see that the enrichment *P*-value histogram is highly conservative for low-intensity gene sets when differential expression *P*-values are used, and is less influenced by intensity when the log fold change estimate is used instead. Notably, even though we found the per-gene variation to be more intensity dependent in RNA-Seq than in microarray, the intensity dependence of gene sets is similar between the two technologies. Because of this dependence, along with the fact that log fold changes showed a higher correlation between technologies than did *P*-values, we chose to use the estimate of effect size rather than statistical significance to evaluate gene set enrichment.

The results of our gene set analysis of both the RNA-Seq and the microarray data are shown in Supplementary Table S3 and the distributions of the log fold change of some of the most significantly enriched gene sets are shown in Supplementary Figures S8–S10. The enrichment of gene sets for carbohydrate catabolic process and glycolysis in glucose and of mitochondrial respiratory chain and adenosine triphosphate synthesis coupled proton transport serve as confirmation that the experiment captures the difference between the two metabolic states. Two of the three most significantly enriched sets are cytoplasmic translation and mitochondrial translation, for which expression is higher in glucose and in ethanol, respectively. As the rate of respiration in the mitochondria is higher in ethanol than in glucose, this suggests that increase in mitochondrial activity is reflected in a tradeoff of translation from the cytoplasmic ribosomes to the mitochondria. Another notable result is that genes in iron ion homeostasis and ferric-chelate reductase activity are higher expressed in ethanol than in glucose. This is likely due to the important role of iron transport and reduction in heme biosynthesis, which in turn is necessary for the electron transport chain and other respiratory activity (29–31).

We identified higher expression of cytokinesis and cellular budding genes in ethanol, even though growth rate was kept equal between the two samples. Mitochondrial inheritance and distribution is known to be actively regulated by the budding tip and to be necessary for equal and efficient distribution of the organelles (32,33), and indeed some differentially expressed genes in our experiment, such as UTH1, have been identified as being related to both cell wall biogenesis and mitochondrial division (34–36). Our results suggest that this process of mitochondrial inheritance may be transcriptionally regulated in response to the metabolic state or level of respiratory activity.

## DISCUSSION

We demonstrate an experimental and statistical approach for determining the variation added at each stage of a microarray or RNA-Seq experiment. We determined that RNA-Seq shows a greater degree of intensity-dependent variation than do microarrays, with particularly high variance for low-intensity genes and that the intensity-dependent component was contributed mostly by the chip or lane level. With qPCR validation, however, we discovered that microarrays appear to possess some systematic biases in their estimation of differential expression for low-intensity genes. These may result from cross-hybridization between these microarray probes and genes that are affected by the treatment condition. Another possibility is that they result from technical variation induced by the polymerase chain reaction step that is performed in RNA-Seq and qPCR, but not in microarrays. Since this bias appears to be consistent across biological, technical and chip replicates, it likely cannot be solved or even detected by performing additional replicates on the microarray platform.

Our results have implications for the design of microarray and RNA-Seq experiments meant to identify differential expression. While other experiments will vary in the amount of variation added at the biological stages, that variation is likely to be intensity-independent as it was in our study, meaning our qualitative conclusions are likely to hold. For high-intensity genes there is little difference either in the genes called significant or the estimate of effect size between RNA-Seq and microarray, and therefore the decision of which technology can be made on other criteria, such as cost. However, in low-intensity genes, the RNA-Seq technology tends to add greater variation, leading to lower statistical power and greater uncertainty in expression estimates. Microarrays, while more consistent in their estimates across technical replicates, may show systematic biases at low intensities that confound differential expression detection. This suggests that studies for which low-expressed genes are of special interest should be performed cross-platform.

More importantly, our study has demonstrated that the intensity-dependent nature of variation must be taken into account in future technology comparisons and quality control experiments, and that focusing on log-fold change estimate agreement rather than significance testing leads to more consistent conclusions. Our approaches of dividing genes into intensity bins, observing correlations within overlapping windows, and smoothing per-gene values using LOESS showed trends and differences in the technologies that would have been missed using aggregate statistics. Finally, we have demonstrated how our experimental data is an appropriate benchmark for comparing statistical analysis methods and for developing experimental recommendations, as it analyzes a well-studied system, includes variation at each stage of an experiment, and compares RNA-Seq and microarrays directly on the same biological samples. We expect future research by ourselves and others will extend our conclusions and develop them further.

## ACCESSION NUMBERS

The microarray data are available from the Gene Expression Omnibus (accession GSE65365) and the RNA-Seq reads are available from the NCBI Short Read Archive (BioProject accession PRJNA271248).

## SUPPLEMENTARY DATA

Supplementary Data are available at NAR Online.

SUPPLEMENTARY DATA

## References

[1] ’t Hoen P.A., Ariyurek Y., Thygesen H.H., Vreugdenhil E., Vossen R.H., de Menezes R.X., Boer J.M., van Ommen G.J., den Dunnen J.T. (2008). Deep sequencing-based expression analysis shows major advances in robustness, resolution and inter-lab portability over five microarray platforms. Nucleic Acids Res..

[2] Zhao S., Fung-Leung W.-P., Bittner A., Ngo K., Liu X. (2014). Comparison of RNA-Seq and microarray in transcriptome profiling of activated T cells. PLoS One.

[3] Willenbrock H., Salomon J., Skilde R., Barken K.B., Hansen T.N., Nielsen F.C., Mller S., Litman T. (2009). Quantitative miRNA expression analysis: comparing microarrays with next-generation sequencing. RNA.

[4] McIntyre L.M., Lopiano K.K., Morse A.M., Amin V., Oberg A.L., Young L.J., Nuzhdin S.V. (2011). RNA-seq: technical variability and sampling. BMC Genomics.

[5] Novak J.P., Sladek R., Hudson T.J. (2002). Characterization of variability in large-scale gene expression data: implications for study design. Genomics.

[6] Tu Y., Stolovitzky G., Klein U. (2002). Quantitative noise analysis for gene expression microarray experiments. Proc. Natl. Acad. Sci. U.S.A..

[7] Labaj P.P., Leparc G.G., Linggi B.E., Markillie L.M., Wiley H.S., Kreil D.P. (2011). Characterization and improvement of RNA-Seq precision in quantitative transcript expression profiling. Bioinformatics.

[8] Tarazona S., Garca-Alcalde F., Dopazo J., Ferrer A., Conesa A. (2011). Differential expression in RNA-seq: a matter of depth. Genome Res..

[9] Wang C., Gong B., Bushel P.R., Thierry-Mieg J., Thierry-Mieg D., Xu J., Fang H., Hong H., Shen J., Su Z. (2014). The concordance between RNA-seq and microarray data depends on chemical treatment and transcript abundance. Nat. Biotechnol..

[10] Bullard J.H., Purdom E., Hansen K.D. (2010). Evaluation of statistical methods for normalization and differential expression in mRNA-Seq experiments. BMC Genomics.

[11] Law C.W., Chen Y., Shi W., Smyth G.K. (2014). Voom: precision weights unlock linear model analysis tools for RNA-seq read counts. Genome Biol..

[12] Hurlbert S.H. (1984). Pseudoreplication and the design of ecological field experiments. Ecol. Monogr..

[13] Smyth G.K., Gentleman R, Carey V, Dudoit S, Irizarry R, Huber W (2005). Limma: linear models for microarray data. Bioinformatics and Computational Biology Solutions using R and Bioconductor.

[14] Robinson M., Oshlack A. (2010). A scaling normalization method for differential expression analysis of RNA-seq data. Genome Biol..

[15] Anders S., Huber W. (2010). Differential expression analysis for sequence count data. Genome Biol..

[16] Novick A., Szilard L. (1950). Description of the chemostat. Science.

[17] Kolkman A., Olsthoorn M., Heeremans C. (2005). Comparative proteome analysis of Saccharomyces cerevisiae grown in chemostat cultures limited for glucose or ethanol. Mol. Cell. Proteomics.

[18] Storey J.D., Tibshirani R. (2003). Statistical significance for genome-wide studies. Proc. Natl. Acad. Sci. U.S.A..

[19] Cameron A.C., Windmeijer F. (1997). An R-squared measure of goodness of fit for some common nonlinear regression models. J. Econometrics.

[20] Love M.I., Huber W., Anders S. (2014). Moderated estimation of fold change and dispersion for RNA-seq data with DESeq2. Genome Biol..

[21] Ambroise J., Bearzatto B., Robert A., Govaerts B., Macq B., Gala J.-L. (2011). Impact of the spotted microarray preprocessing method on fold-change compression and variance stability. BMC Bioinformatics.

[22] Subramanian A., Tamayo P., Mootha V.K., Mukherjee S., Ebert B.L., Gillette M.A., Paulovich A., Pomeroy S.L., Golub T.R., Lander E.S. (2005). Gene set enrichment analysis: a knowledge-based approach for interpreting genome-wide expression profiles. Proc. Natl. Acad. Sci. U.SA..

[23] Ackermann M., Strimmer K. (2009). A general modular framework for gene set enrichment analysis. BMC Bioinformatics.

[24] Gresham D., Boer V.M., Caudy A., Ziv N., Brandt N.J., Storey J.D., Botstein D. (2011). System-level analysis of genes and functions affecting survival during nutrient starvation in Saccharomyces cerevisiae. Genetics.

[25] Hung J.-H., Yang T.-H., Hu Z., Weng Z., Delisi C. (2012). Gene set enrichment analysis: performance evaluation and usage guidelines. Brief. Bioinform..

[26] Oshlack A., Wakefield M.J. (2009). Transcript length bias in RNA-seq data confounds systems biology. Biol. Direct..

[27] Gao L., Fang Z., Zhang K., Zhi D. (2011). Length bias correction for RNA-seq data in gene set analyses. Bioinformatics.

[28] Robinson D.G., Chen W., Storey J.D., Gresham D. (2014). Design and analysis of Bar-seq experiments. G3: Genes | Genomes | Genetics.

[29] Outten C.E., Albetel A.-N. (2013). Iron sensing and regulation in Saccharomyces cerevisiae: ironing out the mechanistic details. Curr. Opin. Microbiol..

[30] Morano K.A., Grant C.M., Moye-Rowley W.S. (2012). The response to heat shock and oxidative stress in Saccharomyces cerevisiae. Genetics.

[31] Lange H. (1999). Mechanism of iron transport to the site of heme synthesis inside yeast mitochondria. J. Biol. Chem..

[32] Simon V.R., Karmon S.L., Pon L.A. (1997). Mitochondrial inheritance: cell cycle and actin cable dependence of polarized mitochondrial movements in Saccharomyces cerevisiae. Cell Motil. Cytoskel..

[33] Boldogh I.R., Fehrenbacher K.L., Yang H.-C., Pon L.A. (2005). Mitochondrial movement and inheritance in budding yeast. Gene.

[34] Camougrand N.M., Mouassite M., Velours G.M., Gurin M.G. (2000). The ‘SUN’ Family: UTH1, an ageing gene, is also involved in the regulation of mitochondria biogenesis in Saccharomyces cerevisiae. Arch. Biochem. Biophys..

[35] Velours G., Boucheron C., Manon S., Camougrand N. (2002). Dual cell wall/mitochondria localization of the SUN family proteins. FEMS Microbiol. Lett..

[36] Camougrand N., Kiov I., Velours G., Manon S. (2004). Uth1p: a yeast mitochondrial protein at the crossroads of stress, degradation and cell death. FEMS Yeast Res..

