# Supplementary material for: A nested parallel experiment demonstrates differences in intensity-dependence between RNA-seq and microarrays
Source: Nucleic Acids Res. 2015 Jun 30;43(20):e131. doi: 10.1093/nar/gkv636 (PMC4787771; doi:10.1093/nar/gkv636)
Supplement: SUPPLEMENTARY DATA [file supp_43_20_e131__index.html]

A nested parallel experiment demonstrates differences in intensity-dependence between RNA-seq and microarrays — SUPPLEMENTARY DATA 

# A nested parallel experiment demonstrates differences in intensity-dependence between RNA-seq and microarrays

## SUPPLEMENTARY DATA

- SUPPLEMENTARY DATA
- SUPPLEMENTARY DATA
- SUPPLEMENTARY DATA
