# Supplementary material for: A nested parallel experiment demonstrates differences in intensity-dependence between RNA-seq and microarrays
Source: Nucleic Acids Res. 2015 Jun 30;43(20):e131. doi: 10.1093/nar/gkv636 (PMC4787771; doi:10.1093/nar/gkv636)
Supplement: SUPPLEMENTARY DATA [file supp_gkv636_nar-00754-met-g-2015-File002.pdf]

Supplementary Information:
A Nested Parallel Experiment Demonstrates Differences in Intensity-Dependence Between RNA-Seq and Microarrays

David G. Robinson, Jean Y. Wang, and John D. Storey[1]

Supplementary Methods

Experimental Protocol

- Details of the media used can be found at http://www.princeton.edu/genomics/botstein/protocols/Dex_lim.htm.
- Details of the protocol for harvesting RNA from the chemostat can be found at http://dunham.gs.washington.edu/MDchemostat.pdf.
- The R64 release of the *S. cerevisiae* genome is available at http://downloads.yeastgenome.org/sequence/S288C_reference/genome_releases/.

Percent of Variance Explained

Percent of variance explained by each level was computed by using ANOVA to compare three nested linear models. The three methods represented, respectively, treatment, treament × biological, and treatment × biological × preparation. Each takes the form

$$Y = \beta {X_{1:p+1}}^T + E$$

where

- p is the number of degrees of freedom used by the parameters in the model (respectively 1, 3, and 7 for the three models)
- g is the number of genes
- Y is the $g \times 16$ matrix of red/green fold changes in microarray data or the log-transformed counts of RNA-Seq data
- $X_{1:p+1}$ is the first $p+1$ columns of the experimental design matrix (Figure S11), which includes the intercept and the p terms fit in the model
- β is the $g \times (p+1)$ matrix of terms
- E is the $g \times 16$ matrix of normally distributed residuals

An adjusted R^2 was found for each of the models ($1 - \frac{RSS(16-1)}{TSS(16-p-1)}$), as shown:

Model	p	Adjusted R^2
Treatment	1	R^2_T
Treatment×Biological	3	R^2_B
Treatment×Biological×Preparation	7	R^2_P

[1]To whom correspondence should be addressed. Email: jstorey@princeton.edu

The percent of variation explained by each level was reported for each gene as R^2_T for the treatment variation, $R^2_B - R^2_T$ for the biological variation, $R^2_P - R^2_B$ for the preparation variation, and $1 - R^2_P$ for the residual variation that occurs on the chip or the lane. This metric is meant to break down the amount that the quantification of expression, and therefore the differential expression estimation, is affected by each level.

Since RNA-Seq data consists of counts rather than continuous measurements, we considered the possibility that the linear model, and therefore the R^2 measure, might not capture the distribution of variation correctly. We thus also calculated two alternative R^2 generalized across exponential families: $R^2_{DEV,P}$ which assumes a Poisson model for the residual noise, and $R^2_{DEV,NB2(ML)}$, which assumes a negative binomial model [1]. This metric is defined as the decrease in Kullback-Leibler divergence between the observations and the expectation under the full model versus the divergence between the observations and the expectation under a null model. The standard R^2 used in linear models with normal residuals can be viewed as a special case of this metric, so this divergence is a useful choice for comparing the count values to the traditional R^2 used in the microarray [2].

We used the edgeR package to perform a negative binomial fit on each gene and estimate the dispersion parameter. We define $y_{i,j}$ as the unnormalized count of gene i in sample j, $\hat{\mu_{i,j}}$ as the expected value of $y_{i,j}$ under the full model (using `fitted` on an edgeR fit), $\bar{y}_{i,j}$ as the expected value using only an intercept term and the library size normalization, $\hat{\alpha_i}$ is the dispersion of gene i estimated using edgeR, n is the number of samples (16) and p is the number of parameters estimated by the model (1 for the condition effect, 3 for condition×biological, and 7 for condition×biological×preparation). The alternative R^2 estimates for the Poisson and negative binomial models can then be calculated as

$$R^2_{DEV,P,i} = 1 - \frac{\sum_{j=1}^{N} y_{i,j}\log(y_{i,j}/\hat{\mu}_{i,j}) - (y_{i,j} - \bar{\mu}_{i,j})}{\sum_{j=1}^{N} y_{i,j}\log(y_{i,j}/\bar{y}_{i,j}) - (y_{i,j} - \bar{y}_{i,j})}\frac{n-1}{n-p-1},$$

$$R^2_{DEV,NB2(ML),i} = 1 - \frac{\sum_{j=1}^{N} y_{i,j}\log(\hat{y_{i,j}}/\hat{\mu}_{i,j}) - (y_i + \hat{\phi}_i^{-1})\log(\frac{y_{i,j}+\hat{\phi}_i^{-1}}{\hat{\mu}_{i,j}+\hat{\phi}_i^{-1}})}{\sum_{j=1}^{N} y_{i,j}\log(\hat{y_{i,j}}/\bar{y}_{i,j}) - (y_i + \hat{\hat{\phi}_i^{-1}})\log(\frac{y_{i,j}+\hat{\phi}_i^{-1}}{\bar{y}_{i,j}+\hat{\phi}_i^{-1}})}\frac{n-1}{n-p-1}.$$

The same three models fit in the traditional linear model were fit using Poisson or negative binomial regression, using edgeR's implementation:

$$Y_{i,\cdot} \sim \text{Pois}(\exp(\beta_i X_{i,1:p+1}{}^T))$$

$$Y_{i,\cdot} \sim \text{NegBin}(\exp(\beta_i X_{i,1:p+1}{}^T)), \alpha_i)$$

then their respective R^2_T, R^2_B and R^2_P were calculated for each gene based on the difference in adjusted alternative R^2 between each model. Figure S6 compares the Poisson and negative binomial models to the traditional R^2 on log TMM-normalized counts. TMM was chosen since it is the default used for edgeR normalization, and Figure S5 shows that TMM produces similar results to other normalization methods.

References for Supplementary Methods

1. A C Cameron and Frank Windmeijer. An R-squared measure of goodness of fit for some common nonlinear regression models. *J Econometrics*, 77(2):329–342, 1997.

2. A Colin Cameron and Frank Windmeijer. R-Squared Measures for Count Data Regression Models With Applications to Health-Care Utilization. *J Bus Econ Stat*, 14(2):209–220, 1996.

Supplementary Tables

Sample	Reads
E1B2	5472595
E1A2	6279951
E2A2	7859929
G2A2	8018457
G1A1	9904120
G1B1	9933158
E2B2	11173029
E2B1	11482599
G2A1	11528672
E1A1	11758844
E2A1	12003519
E1B1	12366694
G2B1	12408000
G1A2	13055305
G2B2	13761168
G1B2	17074028

Table S1: Number of mapped reads from each of the 16 samples in the RNA-Seq experiment, ordered by the number of reads.

Table S2 (Table_S2.csv). Differential expression results for genes in *Sacchromyces cerevisiae*, with each row representing one ORF. Columns are **ORF** (systematic ID), **name** and **description**, and then for both microarray (MA) and RNA-Seq (RS) is shown **estimate**, the G/E log2 fold change estimate, **intensity**, the percentile of the average microarray flourescent intensity or the total RNA-Seq read depth, **intensityraw**, the raw value of the intensity or read depth, **p.value**, p-value testing for significant differential expression, **q.value**, q-value calculated for false discovery rate control, and **statistic**, the moderated t-statistic used to test for differential expression.

Table S3 (Table_S3.csv). Gene Ontology (GO) term enrichment p-values, calculated based on a two-sample Wilcoxon rank sum test. Columns are **ID**, the GO term ID, **Term**, the GO term, **Definition**, definition of the GO term, **Count**, number of genes in the experiment that are associated with that GO term, **Ontology**, whether the term falls within the Biological Process (BP), Molecular Function (MF) or Cellular Compartment (CC). Each term has p-values calculated from either the microarray (MA) or RNA-Seq (RS) experiment, using as the metric either the experiment's estimate of log-fold change (estimate) or its p-value for differential expression (pvalue).

Supplementary Figures

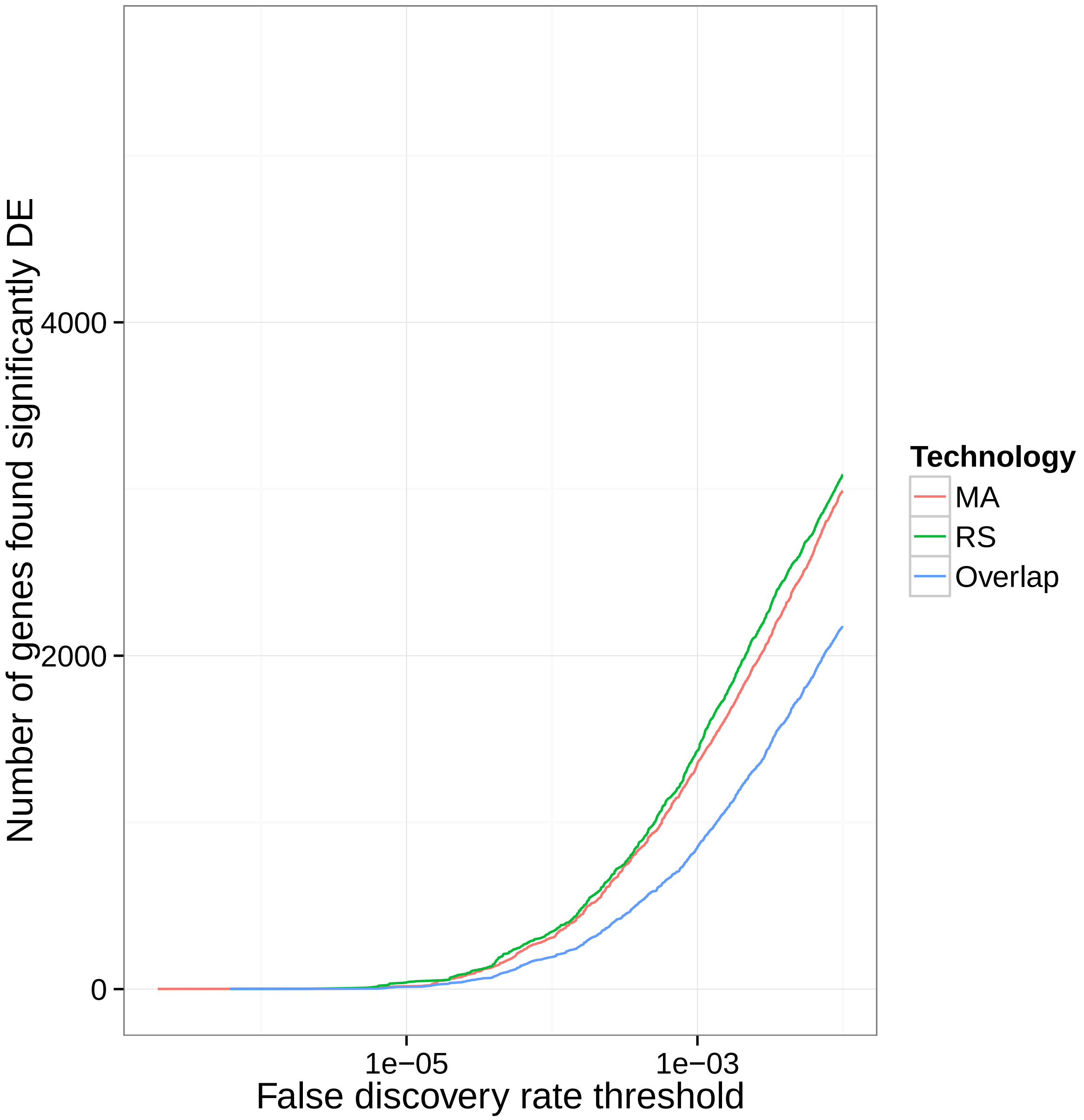

Figure S1: Number of genes found significant differentially expressed in microarray, RNA-Seq, or in both, at various false discovery rate (FDR) thresholds.

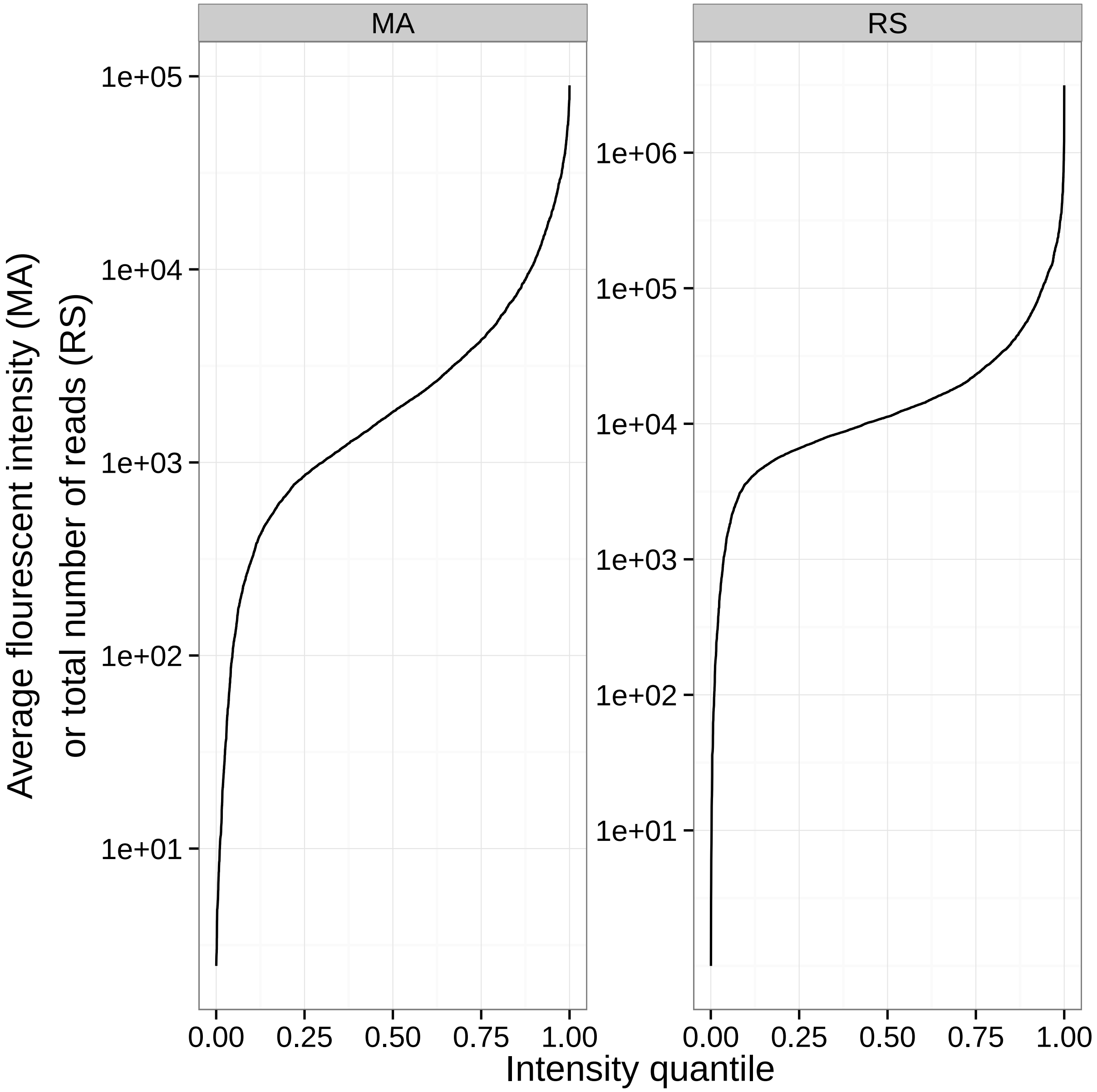

Figure S2: Relationship between the intensity quantile of a gene, as displayed in Figures 2, 3, and 4, to the gene's average flourescence intensity in microarrays or the total number of reads in RNA-Seq.

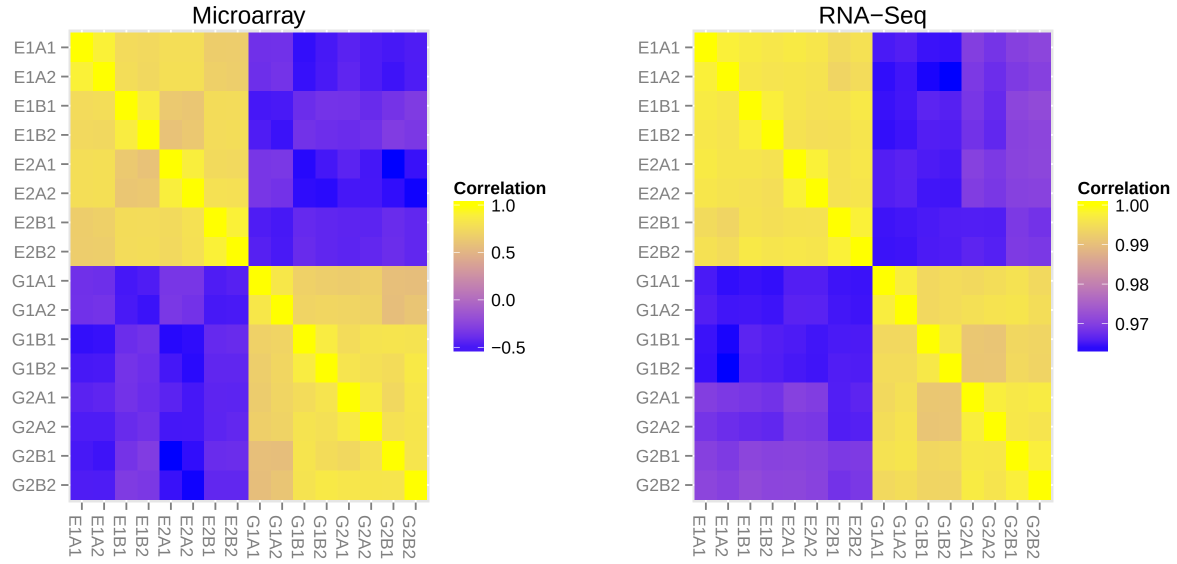

Figure S3: Spearman correlation matrices of microarray and RNA-Seq experiments. The microarray correlation matrix uses the log(cy5/cy3) fold change, where the reference channel is composed of an equally proportioned pool of all the samples, which means the correlation between samples can be negative. The RNA-Seq matrix uses the raw counts, which means the correlation is always close to 1.

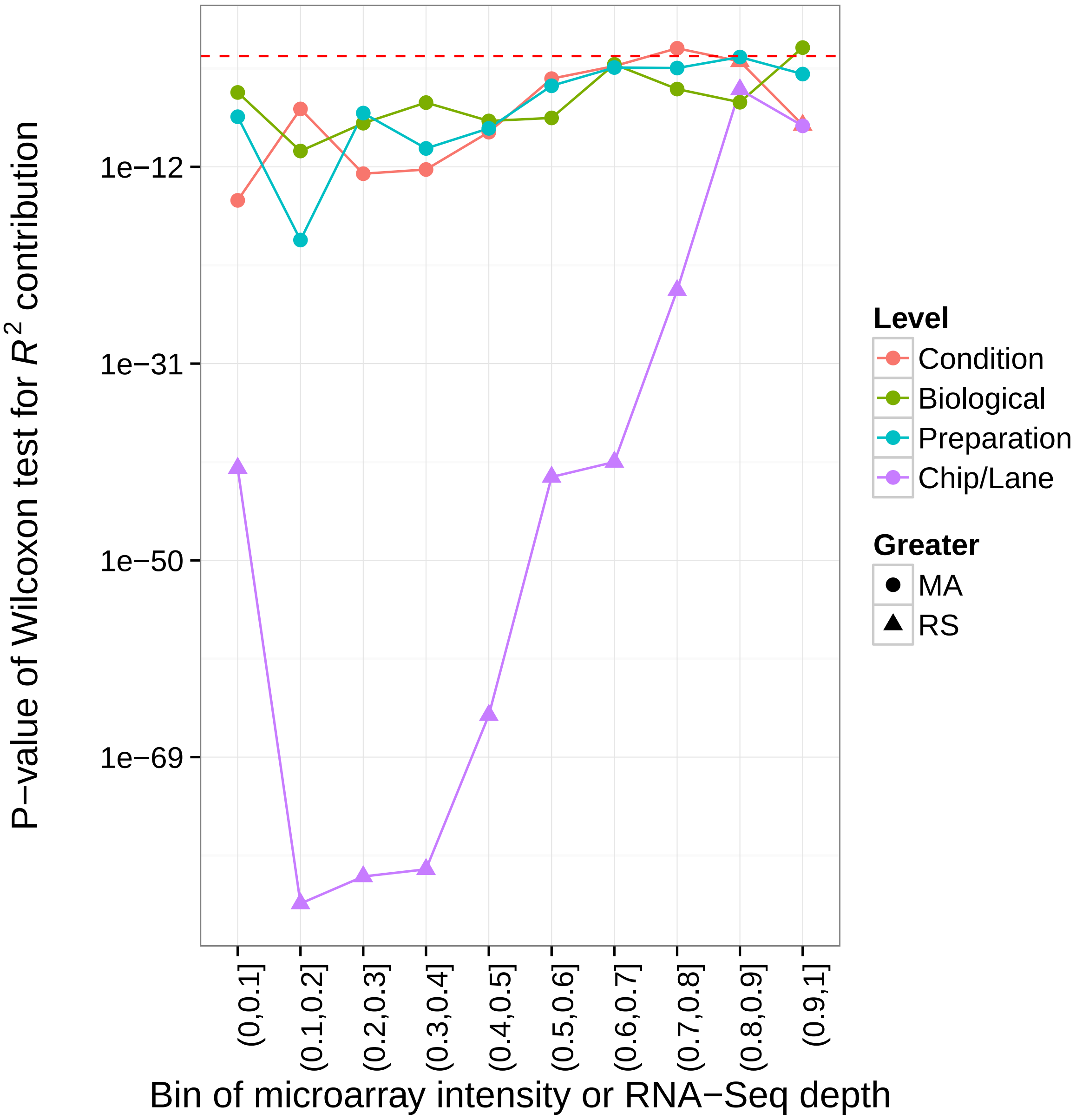

Figure S4: Wilcoxon rank-sum test p-values comparing the percent of variance explained at each level between RNA-Seq and microarrays, in each of 10 bins based on the intensity quantile. The shape of each point indicates whether RNA-Seq or microarrays showed higher R^2 at that stage. The p-values are shown on a log scale, with p = 0.05 shown as a horizontal dashed line.

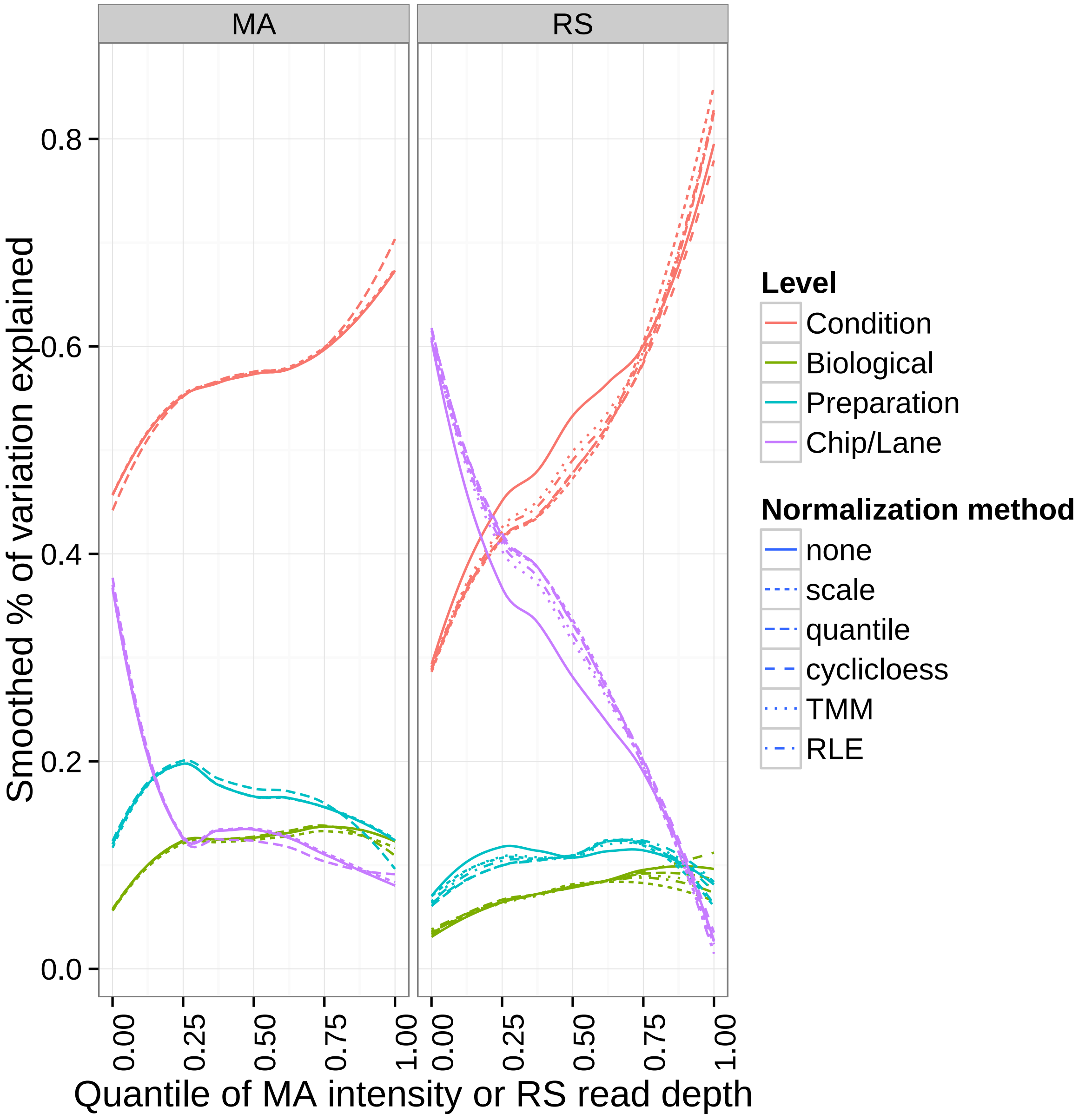

Figure S5: Percent of variance explained by each nested level of the experiment, comparing several normalization methods. "None", "scale", "quantile" and "cyclicloess" denote options used in `normalizeBetweenArrays` in the limma R package, TMM denotes the trimmed mean of M-values method of Robinson and Oshlack 2010, and RLE denotes the "relative log expression" method of Anders and Huber 2010. TMM and RLE were applied only to the RNA-Seq data.

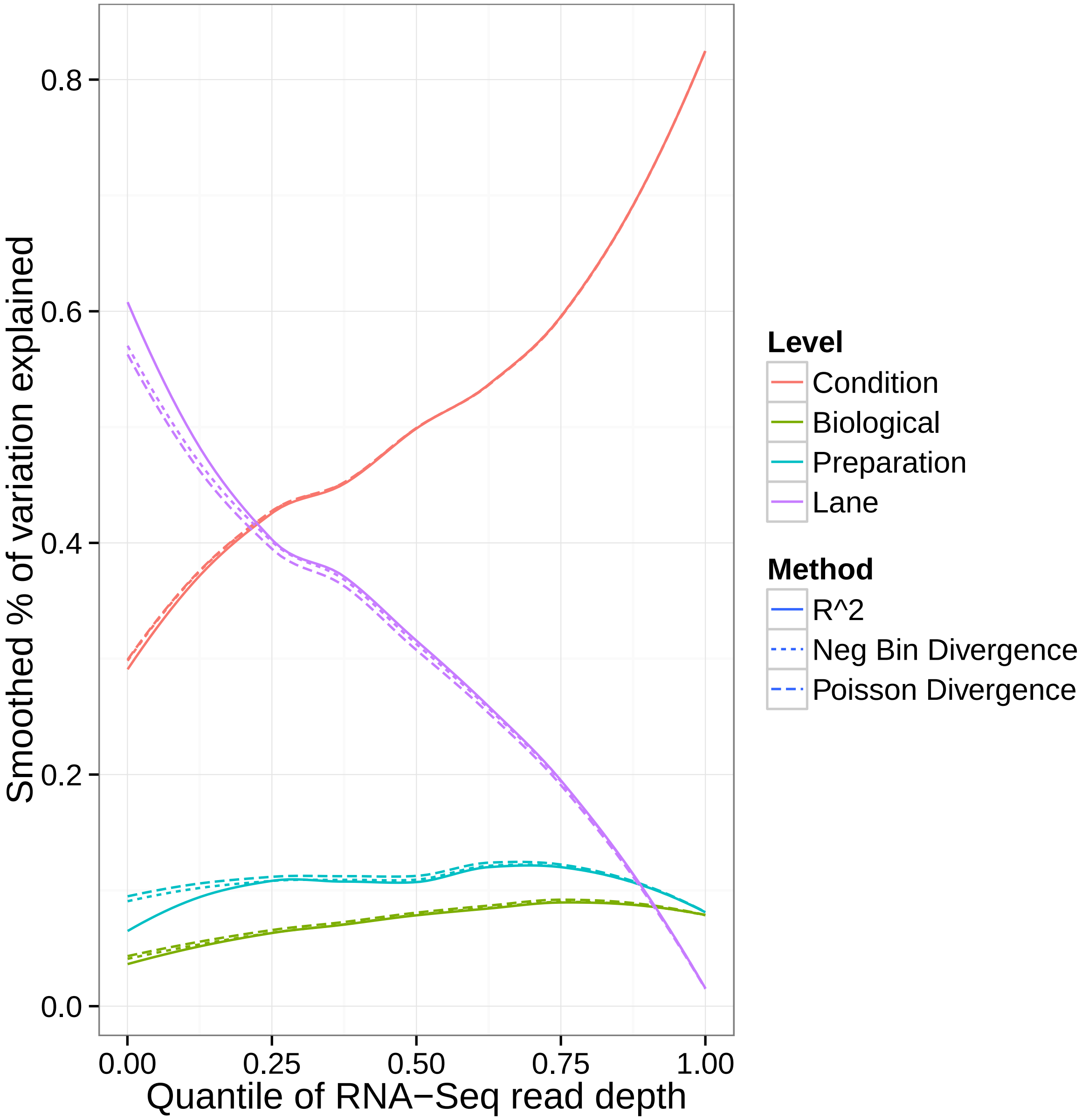

Figure S6: Comparison of an R^2 from ANOVA computed for transformed RNA-Seq, using TMM normalization, to a pseudo-R^2 metric making use of either a Poisson or a negative binomial model.

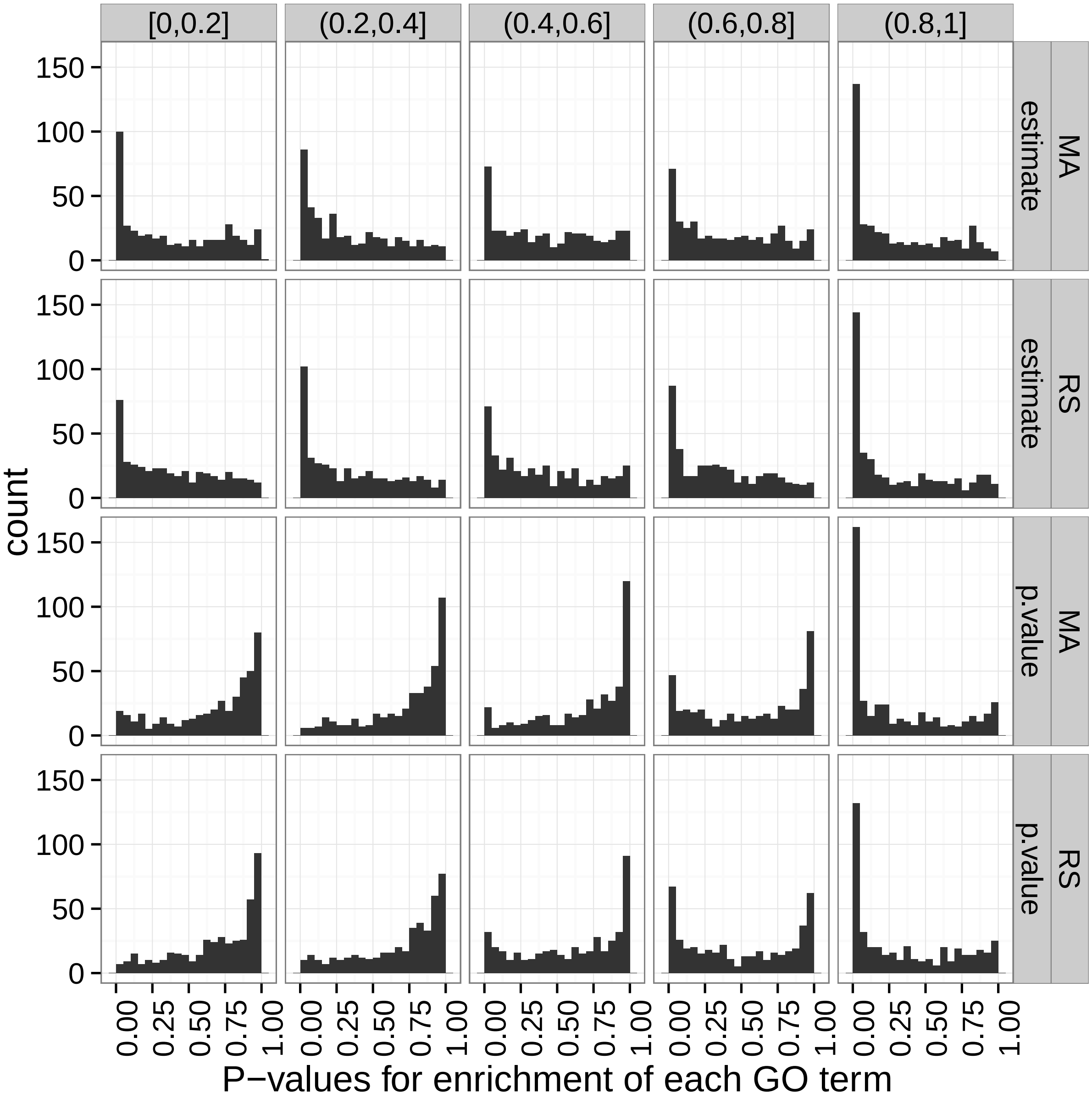

Figure S7: P-value histograms for Wilcoxon tests of gene set enrichment. The plot is divided vertically based on the technology (micrarray or RNA-Seq) and metric (log fold change estimate or p-value) used in the enrichment analysis, and horizontally based on the quantile of the average intensity within each gene.

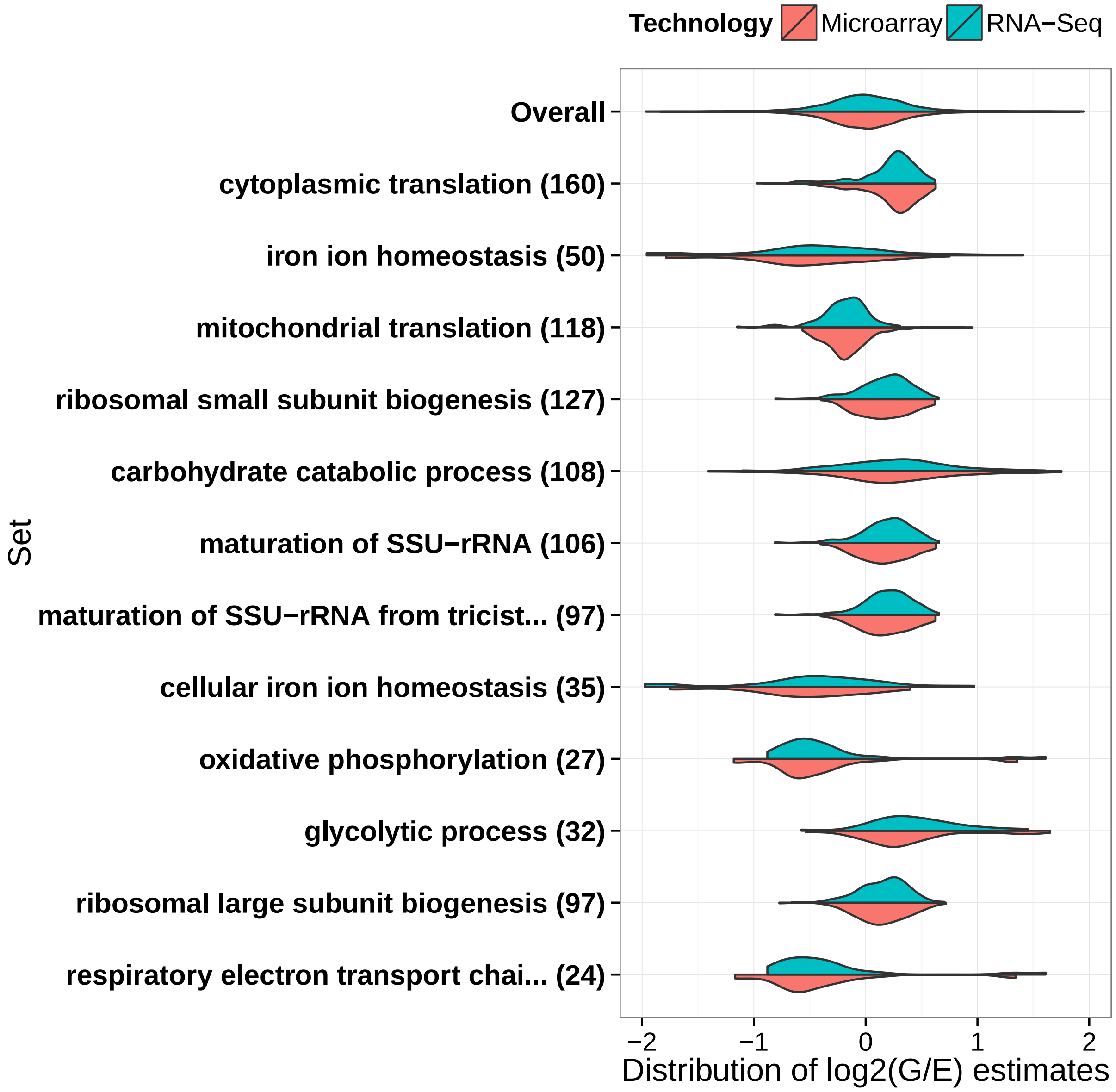

Figure S8: The 12 sets that were found most significantly differentially expressed in microarrays and RNA-Seq, within the Biological Process ontology.

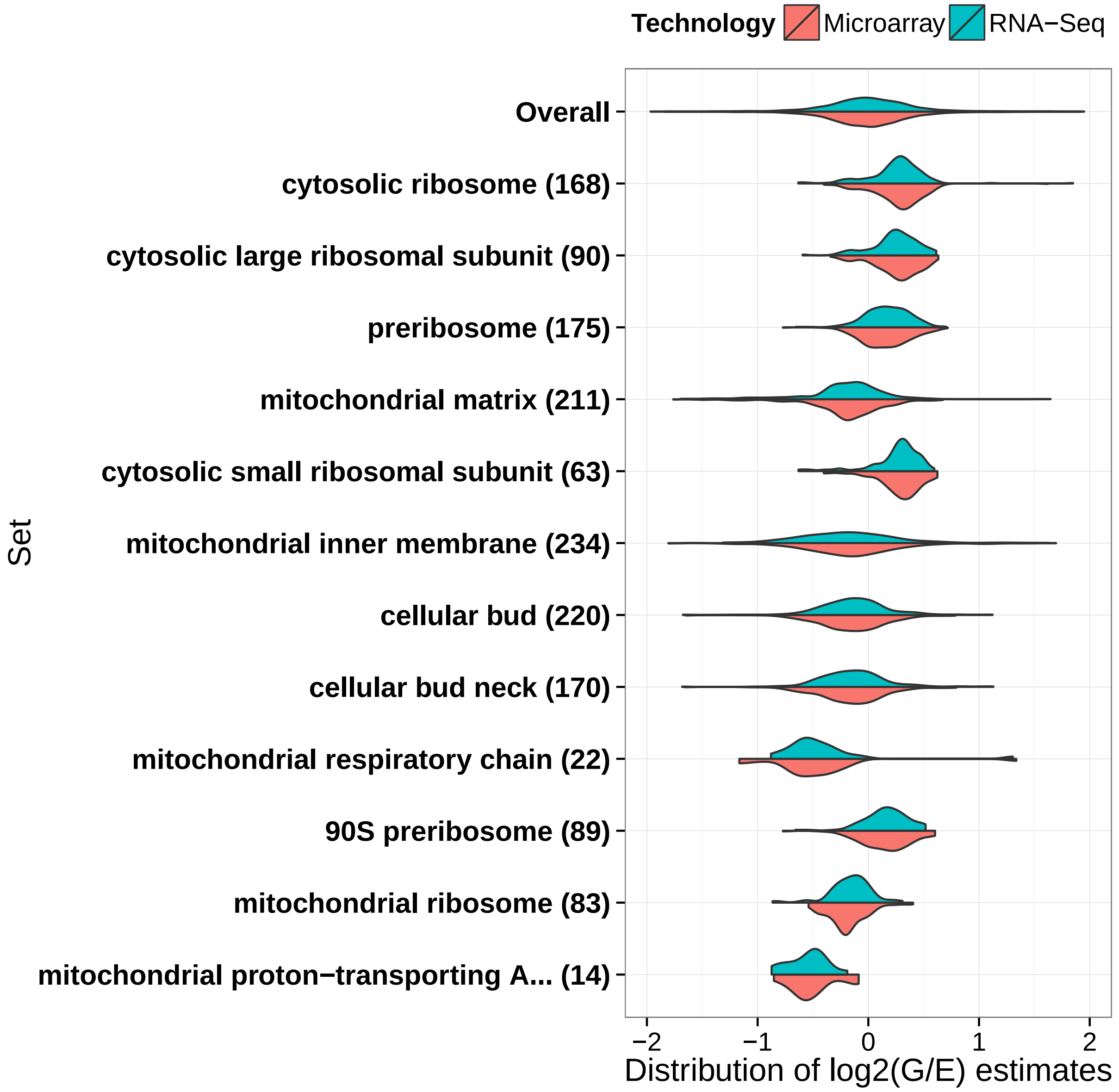

Figure S9: The 12 sets that were found most significantly differentially expressed in microarrays and RNA-Seq, within the Cellular Compartment ontology.

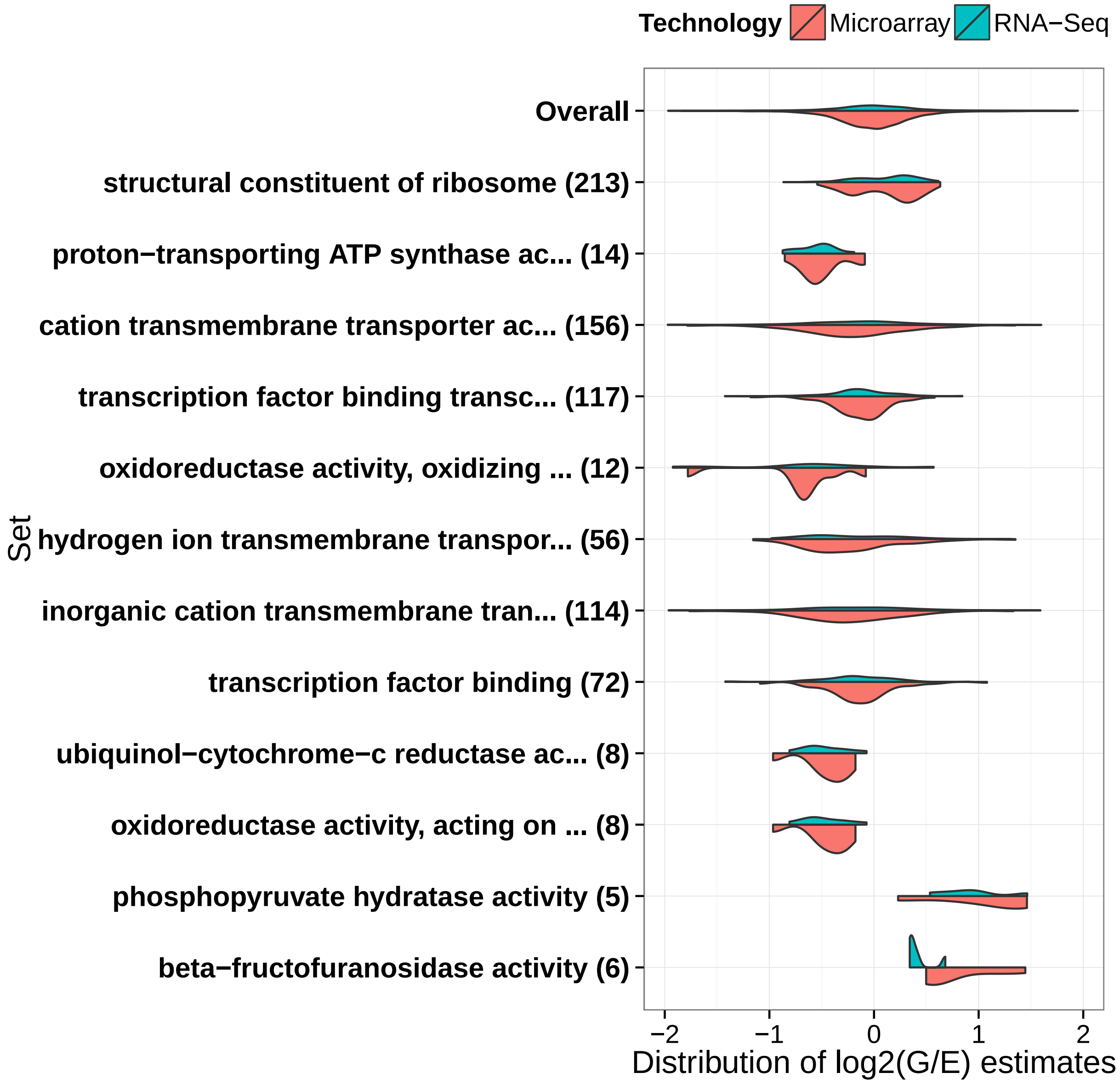

Figure S10: The 12 sets that were found most significantly differentially expressed in microarrays and RNA-Seq, within the Molecular Function ontology.

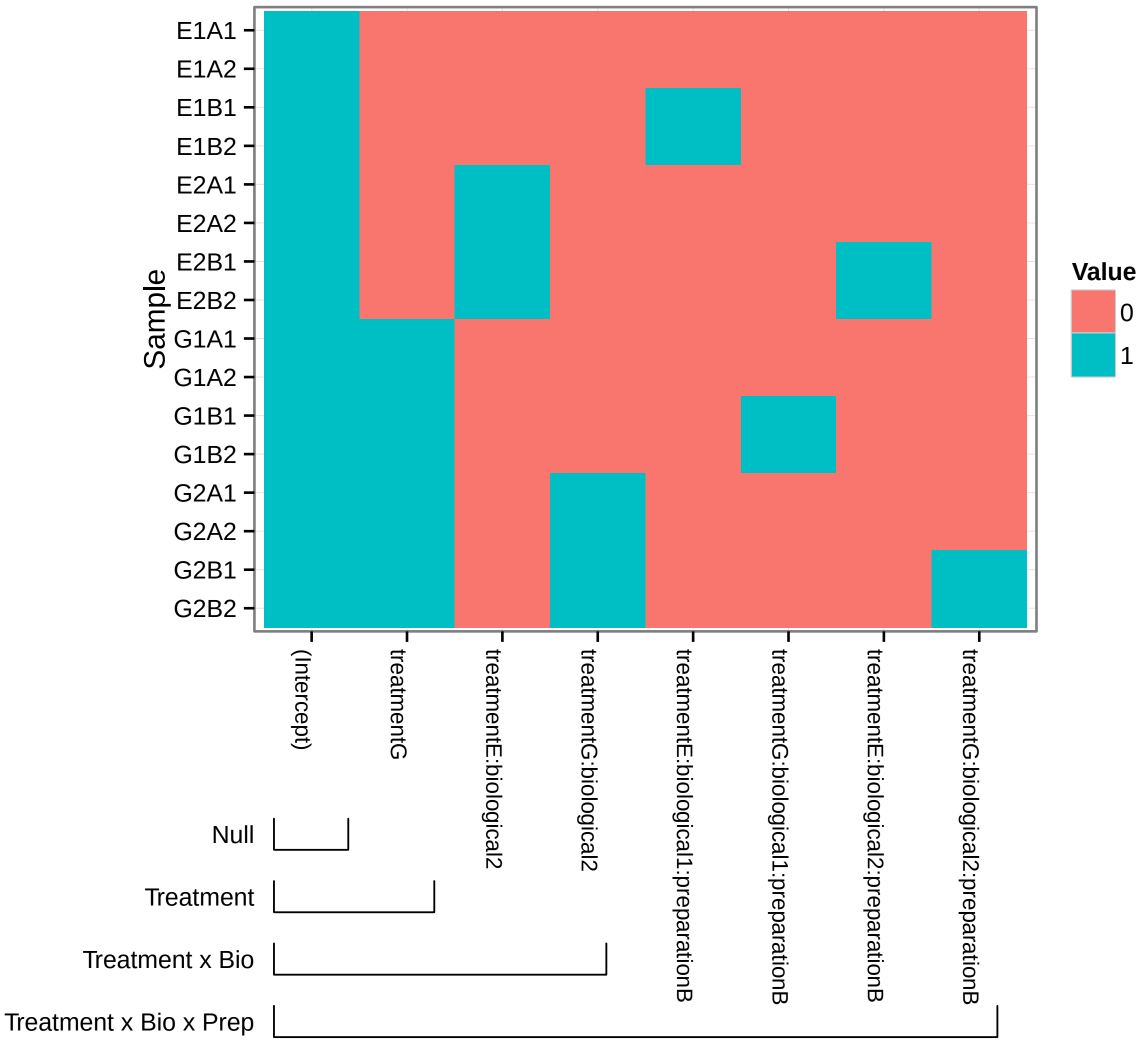

Figure S11: The design matrix X used to fit linear models to the nested experiment and determine R^2. The Null, Treatment, Treatment$\times$Biological, and Treatment$\times$Biological$\times$Preparation models use 1, 2, 4, or 8 columns of this matrix, respectively, as shown.
